# A Novel Class of HIV-1 Inhibitors Targeting the Vpr-Induced G2-Arrest in Macrophages by New Yeast- and Cell-Based High-Throughput Screening

**DOI:** 10.3390/v14061321

**Published:** 2022-06-16

**Authors:** Hirotaka Sato, Tomoyuki Murakami, Ryosuke Matsuura, Masako Abe, Seiji Matsuoka, Yoko Yashiroda, Minoru Yoshida, Hirofumi Akari, Yosuke Nagasawa, Masami Takei, Yoko Aida

**Affiliations:** 1 Viral Infectious Diseases Unit, RIKEN, 2-1 Hirosawa, Wako, Saitama 351-0198, Japan; hirosato@dokkyomed.ac.jp (H.S.); tmurakam@umich.edu (T.M.); matsuura-ryosuke@g.ecc.u-tokyo.ac.jp (R.M.); 2 Division of Hematology and Rheumatology, Department of Medicine, Nihon University School of Medicine, 30-1 Oyaguchi-kamichou, Itabashi-ku, Tokyo 173-8610, Japan; nagasawa.yosuke@nihon-u.ac.jp (Y.N.); takei.masami@nihon-u.ac.jp (M.T.); 3 Department of Microbiology, Dokkyo Medical University School of Medicine, 880 Kitakobayashi, Mibu, Shimotsuga, Tochigi 321-0293, Japan; 4 Laboratory of Global Infectious Diseases Control Science, Graduate School of Agricultural and Life Sciences, The University of Tokyo, 1-1-1 Yayoi, Bunkyo-ku, Tokyo 113-8657, Japan; 5 Drug Discovery Seed Compounds Exploratory Unit, RIKEN Center for Sustainable Resource Science, 2-1 Hirosawa, Wako, Saitama 351-0198, Japan; abe.masako.5e@kyoto-u.ac.jp (M.A.); smatsuoka@riken.jp (S.M.); yoshidam@riken.jp (M.Y.); 6 Chemical Genomics Research Group, RIKEN Center for Sustainable Resource Science, 2-1 Hirosawa, Wako, Saitama 351-0198, Japan; ytyy@riken.jp; 7 Center for the Evolutionary Origins of Human Behavior, Kyoto University, 41-2 Kanrin, Inuyama, Aichi 484-8506, Japan; akari.hirofumi.5z@kyoto-u.ac.jp

**Keywords:** HIV-1, Vpr, HIV-1 inhibitor, G2 arrest, fission yeast, human monocyte-derived macrophages, mammalian cells, high-throughput screening

## Abstract

The human immunodeficiency virus type 1 (HIV-1) accessory protein, Vpr, arrests the cell cycle of the G2 phase, and this Vpr-mediated G2 arrest is implicated in an efficient HIV-1 spread in monocyte-derived macrophages. Here, we screened new candidates for Vpr-targeting HIV-1 inhibitors by using fission yeast- and mammalian cell-based high-throughput screening. First, fission yeast strains expressing the HIV-1 Vpr protein were generated and then treated for 48 h with 20 μM of a synthetic library, including 140,000 chemical compounds. We identified 268 compounds that recovered the growth of Vpr-overexpressing yeast. The selected compounds were then tested in mammalian cells, and those displaying high cytotoxicity were excluded from further cell cycle analysis and imaging-based screening. A flow cytometry analysis confirmed that seven compounds recovered from the Vpr-induced G2 arrest. The cell toxicity and inhibitory effect of HIV-1 replication in human monocyte-derived macrophages (MDM) were examined, and three independent structural compounds, VTD227, VTD232, and VTD263, were able to inhibit HIV-1 replication in MDM. Furthermore, we showed that VTD227, but not VTD232 and VTD263, can directly bind to Vpr. Our results indicate that three new compounds and their derivatives represent new drugs targeting HIV-1 replication and can be potentially used in clinics to improve the current antiretroviral therapy.

## 1. Introduction

The human immunodeficiency virus type 1 (HIV-1) is a lentivirus causing acquired immunodeficiency syndrome (AIDS) in humans. The HIV-1 genome includes not only structural genes, such as *gag*, *pol*, and *env*, but also the accessory genes *tat*, *rev*, *nef*, *vpu*, *vpx*, and *vpr* [1]. Viral protein R (Vpr) is incorporated into the viral particles and is crucial for HIV-1 replication in humans [2,3]. Moreover, Vpr can facilitate viral infection in resting macrophages [4,5,6], but the mechanism by which this occurs is controversial. Vpr is a well-known regulator of HIV-1’s long terminal repeat-driven transcription [7] and is involved in the envelope protein’s expression and virion production [8,9], splicing of mRNA [10,11,12], nuclear import of the viral pre-integration complex [4,13,14], induction of apoptosis [15], trans-activation of the host genes [16,17], and suppression of innate immunity [18]. Vpr’s expression induces an interferon-inducible gene expression [19,20] and modulates the activity of the nuclear factor-κB’s pathway activity [21,22]. The most investigated feature of Vpr is the induction of cell cycle arrest to the G2-phase in infected CD4+ T cells [23,24], among other cell lines [6,25,26]. In addition, it has been previously reported that Vpr-inducing G2 arrest is mediated by the untimely activation of the SLX4 complex [27,28], among other pathways [9,17,29]. The Vpr-dependent G2 arrest is implicated in the efficient HIV-1 spreading in macrophages. Indeed, a mutant of Vpr, defective in the induction of G2 arrest, lacked the ability to promote the HIV-1 infection in the macrophages [8]. This data also suggested that the inhibition of Vpr-inducing G2 arrest is a potent target for the HIV-1 infection in macrophages.

Currently, HIV-1 infected individuals are treated with antiretroviral therapy (ART), which generally includes a cocktail of three to four drugs targeting different steps of the HIV-1 viral life cycle [30,31]. Nevertheless, the use of ART is linked with the development of resistant strains and adverse reactions [31,32,33,34]. Moreover, although ART can reduce viremia and protect against acquired immunodeficiency syndrome’s (AIDS) progression, it was reportedly unable to completely eradicate viruses from the body. Indeed, during ART, the HIV genome persists in non-dividing myeloid cells, such as macrophages [35,36,37]. Macrophages are an early cellular target for the HIV-1 infection, and the transmission between humans is caused by the macrophage-tropic virus rather than the CD4+ T cell-tropic virus [38]. In addition, macrophage-tropic HIV-1 is detected during rebound viremia after the discontinuation of ART [39]. Consequently, new and specific therapeutic targets, such as macrophage-targeting treatments, are required for the radical cure of HIV infection [38].

Vpr’s multifunctional activities are attractive targets for the development of HIV therapeutic agents [40,41]. Several drug candidates, such as vipirinin [42], fumagillin [43], hematoxylin and its derivatives [44,45,46,47], and plant extracts [48,49,50], have been previously reported to inhibit HIV-1 replication via binding to the Vpr protein and/or by interrupting the ability of Vpr, such as through viral gene expression and the nuclear transport of the pre-integration complex.

In this study, we employed previously described Vpr-expressing yeast as a tool for screening the inhibitors of the Vpr-mediated G2/M cell cycle arrest [43,51]. Small compounds were explored using both fission yeast-based and mammalian cell-based high-throughput screening (HTS). We successfully identified several compounds restraining the Vpr inducing G2-arrest as potential HIV-1 inhibitors. A subsequent analysis in mammalian cells confirmed the antiviral property of three different structurally hit compounds: VTD227, VTD232, and VTD263, with VTD227 directly binding the Vpr protein and inhibiting the virus’ replication in human monocyte-derived macrophages (MDM).

## 2. Materials and Methods

### 2.1. Cell and Yeast

HeLa cells were cultured at 37 °C in 5% CO_2_ in Dulbecco’s Modified Eagle’s Medium (DMEM; Thermo Fisher Scientific, Waltham, MA, USA) supplemented with 10% fetal bovine serum (FBS; Sigma-Aldrich, St. Louis, MO, USA). MDM were differentiated from human CD14+ cells separated from peripheral blood monocytes (PBMCs) collected from healthy donors. Informed consent was obtained from all subjects involved in the study. Differentiation protocol was performed as previously described [4,20,44]. 

YY299 yeast (*h^90^ ade6-M216 leu1-32 ura4-D18 pmd1::ura4 bfr1::ura4*) was chosen for use as a drug-sensitive host strain, as previously described [52].

### 2.2. Plasmids and Chemical Compounds

The following plasmids were used as previously described: the yeast expression vector, pDUAL-FFH1c [53]; the mammalian cellular expression vector, pME18Neo, encoding Flag-tagged HIV Vpr, and its control vector, pME/Flag-Vpr-IRES-ZsGreenI, and pME/Flag-IRES-ZsGreenI [54]; and the glutathione S-transferase (GST) expression vector, pGEX-6P-3, encoding GST-tagged Vpr [55]. 

The chemical compound library for screening was provided by the Drug Discovery Initiative of the University of Tokyo (https://www.ddi.u-tokyo.ac.jp/ (accessed on 1 April 2022)). VTD227 was purchased from Vitas-M (Causeway Bay, Hong Kong), and VTD232 and VTD263 were purchased from Life Chemicals Inc. (Niagara-on-the-lake, ON, Canada) and used in in vitro assay to calculate the 50% inhibitory concentration (IC_50_) and 50% cytotoxic concentration (CC_50_).

### 2.3. Protein Expression and Purification

GST-tagged Vpr protein expression in *E. coli*, and purification using glutathione-sepharose, were performed as previously described [55]. 

### 2.4. Vpr Inhibitor Screening Using Overexpressing Yeast

The HIV *vpr* wild-type gene and R80A mutant were cloned as gateway entry clones by recombining the PCR-amplified fragment from the HIV-1 vector, pNL4-3 (GenBank accession number, M19921). Sequence analysis revealed that the PCR-amplified *vpr* gene lacked any mutations. The *vpr* gene was introduced into the fission yeast expression vector, pDUAL-FFH1c, via recombination to produce the Vpr protein under the control of the thiamine-regulatable *nmt1* promoter [53,56]. For the counter assay, the fission yeast strain, overexpressing the gene encoding human Kelch-like ECH-associated protein 1 (Keap1), was constructed, as above, using the KEAP1 entry clone (a kind gift from Dr. N. Goshima) [57]. Yeast genetic manipulation was performed as previously described [58].

High-throughput drug screening was performed using the WST-1 reagent (Dojindo, Kumamoto, Japan). In this assay, yeast cell growth is measured based on mitochondrial dehydrogenase activity, which cleaves the tetrazolium salt, WST-1, to produce formazan dye. Fission yeast cells, pre-grown on synthetic dextrose (SD) solid medium (Sigma-Aldrich) at 30 °C for 2–3 days, were subsequently grown for 24 h, with vigorous shaking in a minimal medium (MM) (Sigma-Aldrich) containing 10% (*v*/*v*) of SD liquid media to supply a small amount of thiamine. This pre-culture was then diluted 200-fold in MM media and grown at 30 °C for 18 h. Finally, the prepared WST-1 mixture was added to the yeast culture, and the absorbance of the formazan product was measured after incubation for more than 3 h (absorption wavelength, 450 nm; reference wavelength, 650 nm). For the primary high-throughput drug screening, the yeast cells were cultured in 20 μL of MM media containing chemical compounds from the chemical compound library provided by the University of Tokyo at concentrations of 20 μM using 384-well plates, and all chemical compounds and cultures were dispensed using an EDR-384UX multichannel pipette (Biotech, Tokyo, Japan) and a mini-Gene LD-01 single-line dispenser (Biotech).

### 2.5. Cell-Based Cytotoxicity Analysis 

HeLa and MDM cells were cultured in 96-well plates containing the chemical compounds. Next, 10 μL of the Premix WST-1 Cell Proliferation Assay System (TAKARA Bio, Shiga, Japan) was added, and the cells were incubated for 2 h at 37 °C. Next, absorbance at a 450 nm and 690 nm wavelength was measured using an EnSight plate reader (PerkinElmer, Waltham, MA, USA). 

### 2.6. Cell-Based G2 Arrest Inhibition Assay Using CELAVIEW

HeLa cells were transfected with pME/Flag-Vpr-IRES-ZsGreenI using Lipofectamine 2000 (Invitrogen, Carlsbad, CA, USA) following the manufacturer’s instructions, plated in 96-well culture plates, and incubated at 37 °C. At 4 h post-transfection, the compounds were added at 10 μM and incubated for an additional 44 h. The cells were fixed with a 3.6% formaldehyde/phosphate buffer saline (PBS) at room temperature for 10 min and then stained with Hoechst 33342 (Sigma-Aldrich; 20 μg/mL). The cells were analyzed using CELAVIEW RS-100 (Olympus, Tokyo, Japan).

### 2.7. Analysis of the Cell Cycle Using Flow Cytometry

HeLa cells were transfected and treated with the specific compounds as described above. At 48 h post-transfection, the cells were harvested with trypsin/EDTA, incubated in a Na-citrate buffer containing NP-40 (0.1%) and RNase A (10 μg/mL) at 37 °C for 10 min, and then stained with propidium iodide (PI; Sigma-Aldrich) (50 μg/mL). The cells were fixed with 0.5% formaldehyde and analyzed using a FACSCalibur instrument (Becton-Dickinson, Franklin Lakes, NJ, USA) with CELL Quest software (Becton-Dickinson). Ratios of the number of cells in the G1 and G2/M phases (G2 + M: G1 ratios) were calculated using the ModFit LT software (Verity Software House, Topsham, ME, USA).

### 2.8. HIV-1 Virus Preparation

Macrophage tropic NF462 HIV-1 and NF462delVpr stocks were prepared by transfecting pNF462 or pNF462delVpr into HEK293T cells using FuGENE HD (Promega, Madison, WI, USA) for 48 h. Culture supernatants were harvested, and the p24 antigen content was determined by HIV-1 p24 ELISA (Ryukyu Immunology Corporation, Okinawa, Japan). Aliquots of viral stocks were stored at −80 °C.

### 2.9. Inhibition Assay of HIV Replication Treated by Hit Compounds

Anti-HIV-1 activity was determined by infecting MDM cells with HIV-1 NF462 and NF462delVpr virus stock (2 ng Gag p24) for 1 h at 37 °C. Infected cells were washed, replaced with a new medium containing dimethyl sulfoxide (DMSO)/hit compounds, and cultured for eight days. Anti-HIV-1 activity was determined by measuring the p24 antigen in the culture supernatant using an HIV-1 p24 ELISA.

### 2.10. Surface Plasmon Resonance (SPR) Binding Analysis

SPR experiments were performed using a Biacore T200 (GE Healthcare, Chicago, IL, USA). Approximately 8000 response units (RU) of the anti-GST antibody were immobilized on a Series S Sensor Chip CM5 (GE Healthcare) using a standard amine coupling protocol with a GST Capture Kit (GE Healthcare). Typically, 800–1000 RU of GST-Vpr were captured on the chip for each run. A buffer solution (10 mM HEPES pH 7.4, 150 mM NaCl, and 0.005% TWEEN20) containing 5% DMSO was used as the running buffer. Analyte solutions were prepared by adding a DMSO solution of compounds to the buffer to adjust the DMSO concentration to 5%. A solvent correction was performed using a buffer containing 4–6% DMSO.

The binding analysis was conducted at a flow rate of 30 μL/min at 25 °C. In each run, the association phase (60 s) and subsequent dissociation phase (60 s) were monitored. The dissociation constant (Kd) of the analyte was determined from the reference-subtracted sensorgrams by global fitting to a simple 1:1 binding model.

### 2.11. Localization of Vpr Visualized by Confocal Microscopy 

At 4 h post-transfection with pME/Flag-Vpr-IRES-ZsGreenI or pME/Flag-IRES-ZsGreenI, HeLa cells were cultured in the absence or presence of three compounds (VTD227, VTD232, and VTD263) at 10 μM for 44 h. The cells were harvested and subjected to immunofluorescence staining with the mouse anti-FLAG M2 monoclonal antibody (Mab; Sigma-Aldrich), followed by the Alexa Fluor 594-conjugated secondary antibody (Invitrogen). The cellular nuclei were stained by hoechst33342. The stained cells were visualized using an FV-1000 fluorescence microscope (Olympus). 

### 2.12. Expression Level of Vpr Assessed by Western Blotting Analysis

At 4 h post-transfection, with either pME/Flag-Vpr-IRES-ZsGreenI or pME/Flag-IRES-ZsGreenI, the HeLa cells were cultured in the absence or presence of three compounds (VTD227, VTD232, and VTD263) for 44 h. The cells were lysed with a lysis buffer (10 mM Tris-HCl (pH 8.0), 150 mM NaCl, 5 mM EDTA, 1% Triton X-100, and 0.1% SDS) and mixed with a 4× SDS-sample buffer. Proteins were applied to a 15% SDS-polyacrylamide gel and transferred to a polyvinylidene difluoride membrane. The Flag-Vpr protein was detected by anti-FLAG M2 Mab (Sigma-Aldrich), followed by the horseradish peroxidase (HRP)-conjugated goat anti-mouse IgG antibody (Amersham Biosciences, Piscataway, NJ, USA), and human beta-actin was detected by anti-beta-actin Mab, by the HRP-conjugated goat anti-mouse IgG antibody (Amersham Biosciences) and SuperSignal™ West Pico Chemiluminescent Substrate (Thermo Fisher Scientific). Band densities were analyzed by densitometry analysis using ImageJ software (National Institutes of Health, Bethesda, MD, USA).

## 3. Results

### 3.1. First Screening of Vpr Inhibitor Using Yeast-Based High-Throughput Screening

To screen the Vpr inhibitor, we adopted fission yeast, a model where the ability of Vpr to arrest the cell cycle in the G2/M phase was previously reported [59]. In addition, fission yeast grows faster than mammalian cells in vitro. Moreover, this experimental system allows control of the expression of the *vpr* gene via the *nmt1* promoter and thiamine-deficient minimal medium [52].

**Figure 1:**
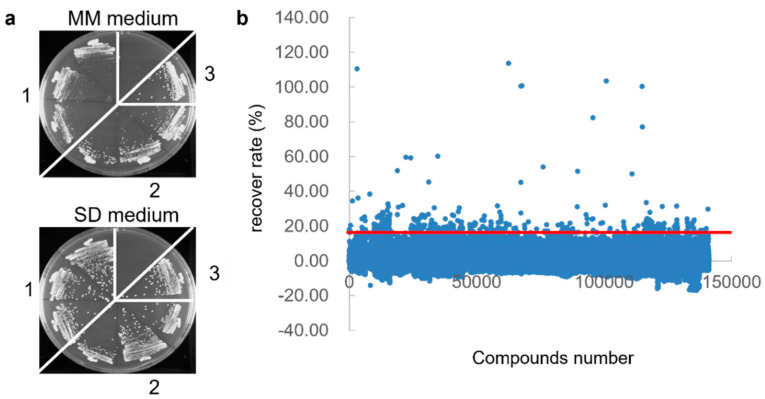
The first screening of the HIV-1 Vpr inhibitor using yeast-based high-throughput screening. (**a**) The growth defect of fission yeast strains with the HIV Vpr overexpression. The fission yeast cells harboring the Vpr-wild type (portion 1) or Vpr mutant R80A (portion 2), or the empty control (portion 3), were streaked on an MM medium for overexpression (upper plate) and an SD medium for repression (lower plate), and they were incubated at 30 °C for 3 days. (**b**) Twenty µM of 140,000 candidate compounds was added to the Vpr-expressing yeast cultured in an MM medium and cultured for 20 h. Yeast cell growth was assessed by WST-1 assay, and the proliferation recovery rate was calculated. The red line represents the hit criteria of the proliferation recovery rate of 15%, which was set as the threshold by over three-fold the standard deviation of all compounds.

First, we constructed fission yeast strains expressing the HIV-1 Vpr protein. We introduced the *vpr* gene that was amplified from the HIV-1 pNL4-3 plasmid by PCR into the yeast expression vector, pDUAL-FFH1c. A drug-sensitive fission yeast strain lacking *pmd1* and *bfr1* [60,61] was transformed with the plasmid and streaked on MM and SD media. The R80A (G2/M arrest defective) mutant Vpr-expressing yeast and empty vector-transformed yeast showed normal growth, whereas the wild-type Vpr-expressing yeast clearly restricted their growth on the MM medium lacking thiamine (Figure 1a, upper plate). This restriction was recovered by culturing the cells in an SD medium (Figure 1a).

Next, the screening was performed, as shown in Table 1. Vpr-overexpressing yeast was cultured in the MM medium for 48 h and treated with 140,000 chemical compounds (20 μM) obtained from the Drug Discovery Initiative at the University of Tokyo. We defined the hit criteria of recovery rate as 15%, indicated by over three-fold the standard deviation of all of the compounds, and found 560 compounds that recovered the growth of Vpr-overexpressing yeast (Figure 1b and Table 1). To confirm the repeatability and dose dependency, we repeated the experiments, using 20, 2, and 0.2 μM of the compounds, twice (Figure S1a and Table S1). Furthermore, to exclude compounds with non-specific and Vpr-independent recovery activity, the cells were treated with the yeast strain overexpressing the human Keap1, a component of E3 ubiquitin ligase [62], under the same conditions as the counter assay (Figure S1b and Table S1). Consequently, only 268 compounds were selected from the 140,000 compounds as hits from the first screening (Table 1). 

### 3.2. Second Screening of Vpr Inhibitor Using HeLa Cell-Based High-Throughput Screening

**Figure 2:**
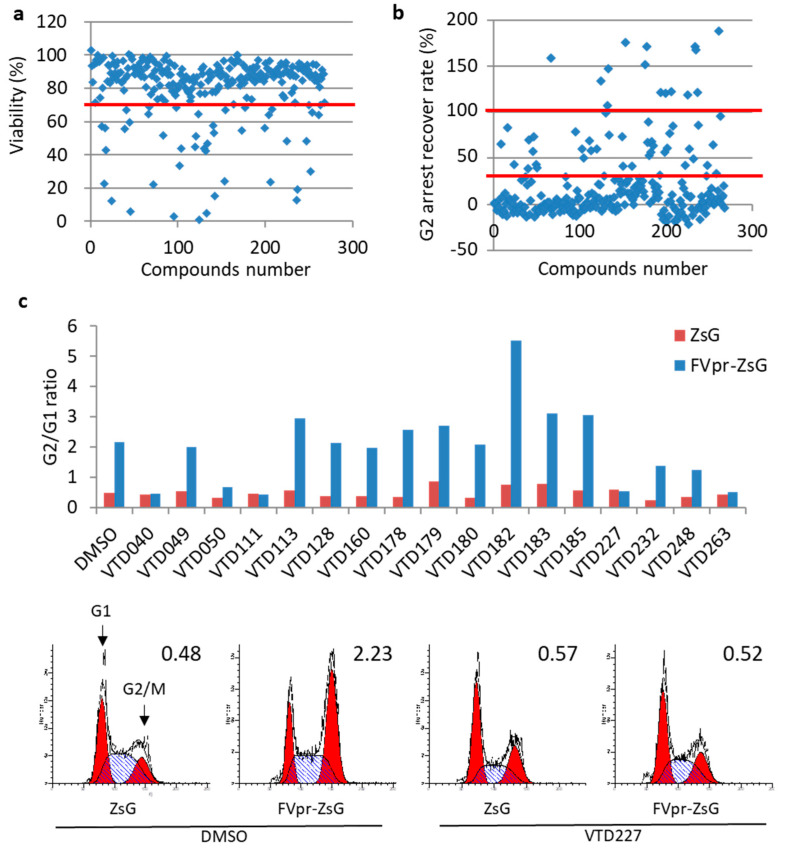
Secondary screening of Vpr inhibitor using HeLa cell-based high-throughput screening. (**a**) HeLa cells were treated with 10 µM of 268 candidate compounds for 48 h, and the effect on the cell viability of each compound was assessed by WST-1 assay. The red line represents the threshold for high toxicity when the treated cells showed viability under 70% compared to the DMSO-treated control. (**b**) The HeLa cells were transfected with pME/Flag-Vpr-IRES-ZsGreenI and incubated at 37 °C. At 4 h post-transfection, 224 candidate compounds were added at 10 µM and incubated for an additional 44 h. The cells were fixed, stained with Hoechst 33342, and analyzed using CELAVIEW RS-100. The red lines represent the threshold for inhibition ranging from over 30% and not exceeding 100% of the G2/M arrest recovery rate. (**c**) The inhibition of the Vpr-induced G2/M cell cycle arrest was confirmed by the flow cytometry. The HeLa cells were transfected with either pME/Flag-Vpr-IRES-ZsGreenI (FVpr-ZsG) or the control pME/Flag-IRES-ZsGreenI (ZsG) and incubated at 37 °C. At 4 h post-transfection, transfected HeLa cells were treated either with 10 µM of 17 candidate compounds or vehicle (DMSO only). After 48 h, the cells were collected and stained with propidium iodide, and the DNA content of ZsGreenI-positive cells was measured by flow cytometry. The cell cycle was analyzed by Modfit software, and the G2/G1 ratio was calculated. Results of the G2/G1 ratio of 17 candidate compounds-treated cells (upper panel) and the typical model of the cell cycle analysis of a representative drug, VTD227 (bottom panel), are shown. Arrowheads indicate peaks of cells at the G1 and G2/M phases. The G2/M:G1 ratio is indicated in the upper right of each graph.

To exclude compounds with high cytotoxicity toward mammalian cells, the HeLa cells were cultured for 48 h with 10 μM of the 268 compounds from the first screening, and cell viability was assessed using the WST-1 assay. We considered the compound highly toxic when the treated cells showed viability under 70% compared to the DMSO-treated control (Figure 2a). The treatment with 224 out of 268 compounds resulted in cell viability above the set threshold (Figure 2a and Table 1).

Next, to determine whether the compounds have inhibitory activity towards Vpr-induced G2/M arrest in mammalian cells, we examined the cell cycle of Vpr-overexpressing HeLa cells using a CELAVIEW cell image analyzer. CELAVIEW RS100 automatically acquires cellular fluorescence images and quantitatively analyzes the morphology and fluorescence signal of a large number of cells. Compared with flow cytometry analysis, CELAVIEW RS100 enables high-throughput analysis of DNA content in Hoechst33342-stained nuclei of large numbers of Vpr-expressing cells. In HeLa cells transfected with either pME/Flag-Vpr-IRES-ZsGreenI or the control pME/Flag-IRES-ZsGreenI, Vpr evidently expressed (Figure S2) and strongly arrested the cell cycle at the G2/M stage after 48 h (Figure 2c). We defined the pME/Flag-Vpr-IRES-ZsGreenI-transfected cells as 100% and the control pME/Flag-IRES-ZsGreenI-transfected cells as 0% of the G2 arrested by Vpr and calculated the recovery rate of a 10 μM compound treatment. Of the total 224, only 17 compounds showed over 30%, and not exceeding 100%, of the G2 arrest recovery rate (Figure 2b and Table 1). Compounds showing a >100% recovery, probably due to the G1 arrest or other cell cycle phenomena, were excluded from further analysis. Furthermore, to confirm whether the candidate compounds recovered the Vpr-induced G2/M arrest, we analyzed the cell cycle of Vpr-expressing HeLa cells upon the addition of 10 μM of candidate compounds using flow cytometry. A treatment with seven compounds, VTD040, VTD050, VTD111, VTD227, VTD232, VTD248, and VTD263, recovered the G2-arrest mediated by Vpr (Figure 2c upper panel and Table 1). Control vector-transfected cells showed a normal cell cycle, and the G2/G1 ratio was 0.48, whereas Vpr-transfected cells clearly arrested their cell cycle in the G2/M-phase, and their G2/G1 ratio was 2.23 (Figure 2c, bottom panel). In contrast, after treatment with the representative drug, VTD227, among the seven compounds, the G2/M:G1 ratio for the pME/Flag-Vpr-IRES-ZsGreenI-transfected cells decreased to 0.52, which was the same as that of the control vector-transfected cells (G2/M:G1 ratio = 0.57), indicating that VTD227 successfully inhibited Vpr-induced G2/M-phase cell cycle arrest. In addition, immune fluorescence analyses and Western blotting analyses showed that the localization and expression level of Vpr in the HeLa cells were not affected by three (VTD227, VTD232, and VTD263) out of seven representative compounds, 44 h post-treatment (Figures S3 and S4). 

### 3.3. Third Screening of Vpr Inhibitor Using Human Monocyte-Derived Macrophages (MDM)

**Figure 3:**
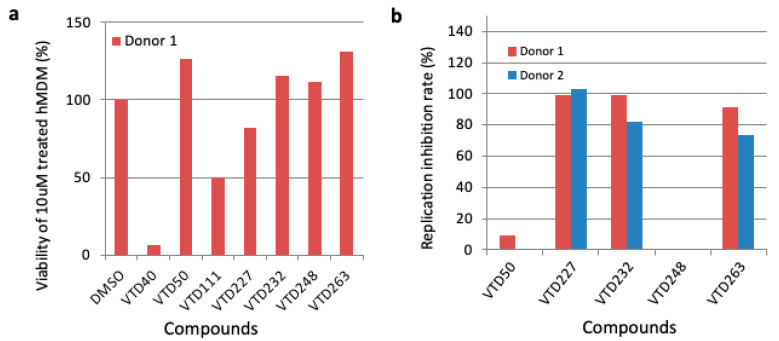
Third screening of Vpr inhibitor using human monocyte-derived macrophages (MDM). (**a**) Human peripheral blood mononuclear cells (PBMCs) were collected from healthy donor 1, and MDM were differentiated from monocytes isolated from human PBMCs. MDM cells were treated with 10 µM of 7 candidate compounds for 10 days, and the effect on the cell viability of each compound was assessed by a WST-1 assay. (**b**) MDM were differentiated from monocytes isolated from healthy donors 1 and 2, infected with HIV-1 NF462, and then treated with 10 µM of 4 candidate compounds. After 8 days, we collected the supernatant and assessed the inhibitory effect of the compounds on virus replication by p24 ELISA.

In HIV-1-infected patients, macrophages act as a viral reservoir extensively distributed in multiple tissues during ART [63,64]. Importantly, Vpr is crucial for HIV-1 replication in macrophages [4,5,6], and Vpr-mediated G2/M arrest has been implicated in efficient HIV-1 spread in macrophages [8]. Thus, to obtain a compound that suppresses Vpr-mediated G2/M arrest, thereby inhibiting HIV-1 replication in a Vpr-dependent manner in macrophages, we examined the cytotoxicity and effect of the selected seven compounds on the HIV-1 replication in MDM from healthy donors. As shown in Figure 3a, when MDM were treated with the compounds at the concentration of 10 μM, two compounds (VTD40 and VTD111) showed high cytotoxicity towards the MDM obtained from healthy donor 1 (Figure 3a). To confirm the inhibition of HIV-1 replication, we tested the compounds using an HIV-1 p24 capture ELISA. Three compounds, VTD227, VTD232, and VTD263, potently inhibited HIV-1 replication in MDM derived from two healthy donors, 1 and 2 (Figure 3b and Table 2). Due to the research’s ethical restrictions on the number of blood draws allowed in this study, it was not possible to perform statistical analysis in experiments using MDM.

### 3.4. Final Selected Compounds Inhibited the Virus Replication in MDM in a Dose-Dependent Manner

**Figure 4:**
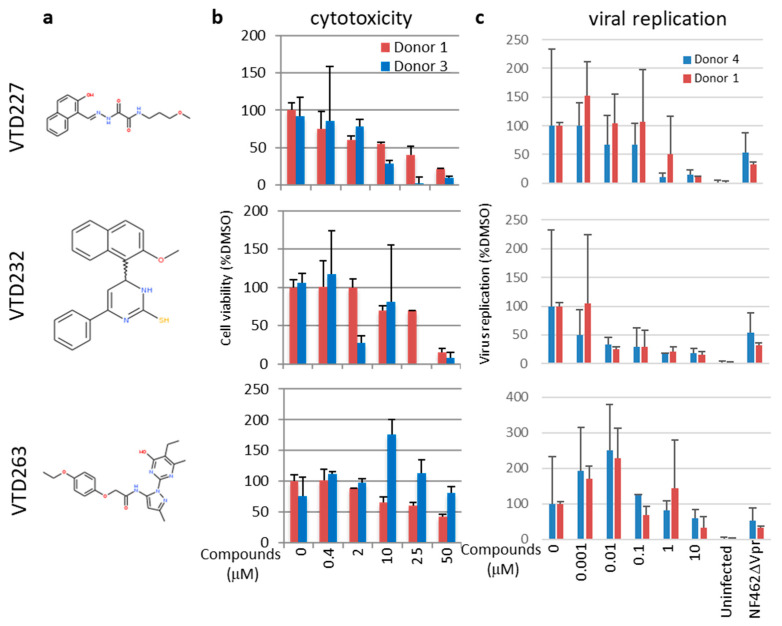
The inhibitory activity of selected compounds on the virus replication in HIV-1 NF462-infected human monocyte-derived macrophages (MDM). (**a**) Structure of the final selected compounds, VTD227, VTD232, and VTD 263. (**b**) Dose-dependent cell cytotoxicity for MDM of the final selected compounds. Differentiated MDM from healthy donors 1 and 3 were treated with 0, 0.4, 2, 10, 25, or 50 µM of 4 candidate compounds for 48 h, and cell viability was assessed by WST-1 assay. (**c**) Inhibition on the replication of HIV-1 NF462 by the final selected compounds. Differentiated MDM from healthy donors 1 and 4 were infected either with NF462 HIV-1 or NF462delVpr and replaced with a new medium containing 0, 0.001, 0.01, 0.1, 1, or 10 µM of 4 candidate compounds. The cells were maintained for 8 days, and the levels of virus production in the culture supernatants were measured by the p24 antigen, ELISA. A calculated 50% replication inhibitory concentration (IC_50_) and 50% cytotoxic concentration (CC_50_) are shown in Table 2. Each column and error bar represent the mean SD of the results from the duplicate samples from two donors.

We identified three effective inhibitor candidates with different structures (Figure 4a). We measured and calculated the concentration of 50% cytotoxicity (CC_50_) and the concentration of 50% inhibition (IC_50_) on the MDM (Figure 4b,c and Table 2). All three compounds inhibited HIV-1 replication in a dose-dependent manner. The CC_50_ of VTD227, VTD232, and VTD263 were 17.9 ± 5.3, 30.5 ± 5.0, and 38.8 ± 0.76 μM, respectively, and their IC_50_ were 0.78 ± 0.59, 0.0039 ± 0.0046, and 11.4 ± 4.0 μM, respectively. Thus, their selective indices (SI; calculated as CC_50_/IC_50_) were 22.9, 7820, and 3.4, respectively.

### 3.5. Directly Binding Availability of Hit Compounds to Vpr Protein

**Figure 5:**
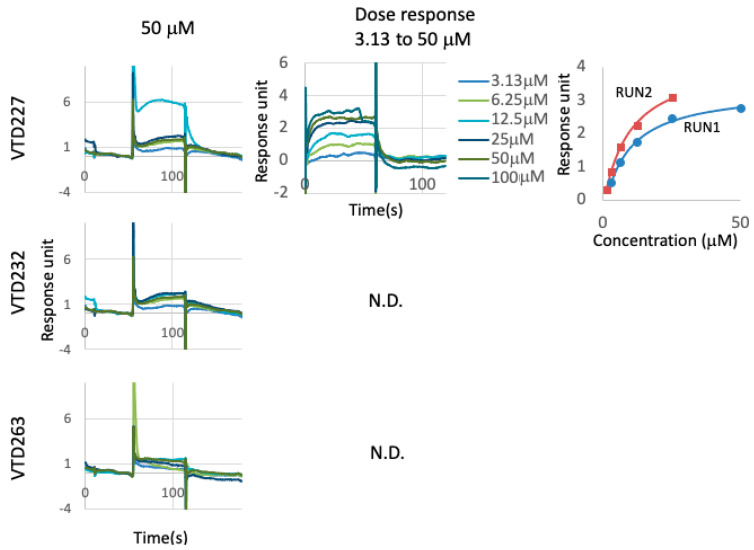
The binding affinity to the Vpr protein of the final selected compounds. To analyze whether the compounds directly bind to the HIV Vpr protein, the binding affinity of the compounds with a purified GST-Vpr protein was assessed by Biacore. GST-Vpr immobilized on the sensor CM5 chips and flew analyte as 50 µM of the compounds, VTD227, VTD232, and VTD263 ((**Left**) panel), and as a dose from 3.13 to 100 µM of the compound, VTD227 ((**Center**) panel). This dissociation constant (Kd) value was calculated by two independent experiments using equilibration analysis ((**Right**) panel), as shown in Table 2. N.D. means as not determined.

To identify the mechanisms behind their inhibitory activity, we analyzed the binding activity of the three identified compounds to the HIV-1 Vpr using surface plasmon resonance (SPR) binding analysis. A purified GST-tagged Vpr protein was immobilized on sensor chips and treated with 50 μM of the compounds; VTD227 was directly bound to Vpr but not to VTD232 and VTD263 (Figure 5). VTD227 also showed binding to Vpr at concentrations ranging from 100 to 3.13 μM in a dose-dependent manner, and the calculated dissociation constant (Kd) was 8.8 ± 0.48 μM (Figure 5 and Table 2). 

## 4. Discussion

Current therapies against HIV-1 infection, including anti-retroviral therapy (ART), fail to completely eliminate viral reservoirs in cells such as macrophages. The HIV-1 Vpr promotes virus production in macrophages, and Vpr-mediated G2/M arrest is implicated in the efficient HIV-1 spreading in these reservoir cells. Thus, a treatment targeting that Vpr-mediated G2/M arrest may represent a useful tool for inhibiting HIV-1 replication in macrophages. Previously, we reported the identification via the chemical array of HIV-1 inhibitors targeting the interaction between Vpr and importin α, such as hematoxylin and its derivatives [44,45,46,47]. In this study, we designed yeast and mammalian cell-based screening systems to study molecules targeting Vpr. Here, HIV-1 potent inhibitors targeting Vpr were selected for their ability to inhibit Vpr-induced G2-arrest. Three such structurally new HIV-1 inhibitor candidates, VTD227, VTD232, and VTD263, were identified by screening a library of 140,000 compounds. These molecules recovered the viability of Vpr-overexpressing yeast, inhibited G2/M arrest induced by Vpr in mammalian cells, and suppressed HIV-1 replication in MDM. Interestingly, the three effective inhibitor candidates are structurally different from one another and from the several previously reported antiviral drugs, such as vipirinin [42], fumagillin [43], hematoxylin and its derivatives [44,45,46,47], and plant extracts [48,49,50]. Thus, the structures and actions of the three selected compounds provide valuable information for the development of a novel class of anti-HIV-I agents in macrophages.

The present study clearly demonstrated that only VTD227 directly interacted with Vpr, whereas VTD232 and VTD263, although presenting an anti-viral phenotype, lack the ability to bind Vpr specifically. Vpr induces G2/M arrest via ubiquitin ligase-dependent and-independent pathways. In the ubiquitination-dependent pathway, Vpr binds to the damage-specific DNA binding protein-1 (DDB1) and forms a ubiquitination complex with cullin4A (CUL4) and DDB1-CUL4-associated factor 1 (DCAF1) [28]. The synthetic lethality of the unknown (X) function 4 (SLX4) is depleted via this pathway, thus inducing cell cycle G2-arrest [28]. Recently, Zhang and Bieniasz reported that CCDC137 is involved in Vpr-inducing G2/M arrest [65]. The Huntingtin-interacting protein 1 (HIP1) was identified as a novel host factor involved in Vpr-induced G2 arrest and HIV-1 infection in macrophages [66]. Furthermore, neither Vpr localization nor its expression level in HeLa cells was altered by the treatment with the three compounds (Figures S3 and S4). Therefore, VTD227 may inhibit G2-arrest by blocking the interaction between the ubiquitination complex and its substrates, such as SLX4 and/or CCDC137, or, alternatively, HIP1. Further studies are needed to confirm the ability of molecules, either here identified or new, to suppress the degradation of such substrates. In contrast, VTD232 and VTD263 did not directly bind to Vpr, and their inhibitory mechanisms remain unclear. VTD263 showed a dual effect on viral replication. Treatment with a high concentration of VTD263 decreased the HIV-1 virus release, whereas 0.01 to 0.001 μM VTD263 tended to increase HIV-1 replication. VTD232 showed strong HIV inhibitory activity at a lower concentration (0.0039 ± 0.0046 μM) and higher SI (7820), thus representing a potent HIV-1 inhibitor candidate. Further investigation of the molecular mechanisms behind the inhibitory effect of these two molecules is required. Finally, it is necessary to clarify whether three compounds inhibit Vpr’s function using macrophage-tropic HIV NF462 viruses encoding a Vpr deletion mutant (ΔVpr).

In this study, we revealed that three potent HIV-1 inhibitors targeting Vpr inhibited not only Vpr-induced G2-arrest but also HIV-1 replication in macrophages. Further experiments are required to analyze the effectiveness of these drugs in long-term treatment for clinical application in HIV-infected MDM after a few rounds of infection to determine whether the inhibition effects of these three compounds are achieved after established infections. However, in addition to macrophages, Vpr enhances HIV-1 infection and HIV-1 gene expression in CD4+ T cells [54,65,66,67,68,69]. Therefore, additional studies are required to determine whether these three compounds could affect the Vpr-mediated enhancement of HIV-1 replication in CD4+ T cells. 

In conclusion, we identified three structurally unrelated compounds able to inhibit HIV-1 replication through a Vpr-dependent mechanism. Furthermore, one compound, VTD227, is a potent inhibitor that acts by directly binding Vpr. Our findings suggest that these molecules may be useful medicinal seeds for the development of new antiviral therapies.

## Figures and Tables

**Table 1:** Screening methods for the new therapeutic drug, targeting HIV Vpr (Tables S1–S3).

Screening Stage	Screening Methods	Compounds’ Number
1st screening	Vpr inhibition on the overexpressing yeast	140,000 to 560
	Reproductivity, dose, and selectivity assay	560 to 268
2nd screening	WST-1 assay (HeLa cell)	268 to 224
	G2 arrest inhibition on the Vpr transfected HeLa cells (CELAVIEW)	224 to 17
	G2 arrest inhibition on the Vpr transfected HeLa cells (flow cytometry)	17 to 7
3rd screening	WST-1 assay (MDM cell)	7 to 5
	HIV replication inhibition on the HIV-infected MDM	5 to 3

**Table 2:** The inhibitory and cytotoxic concentration of final selected compounds.

Compound	IUPAC Name	HeLa ^1^ CC_50_ (µM)	MDM ^2^ CC_50_ (µM)	MDM IC_50_ (µM)	MDM ^3^ SI	Vpr Binding ^4^ Kd (µM)
VTD227	N-[(1E)-2-(2-hydroxynaphthyl)-1-azavinyl]-N′-(3-methoxypropyl)ethane-1,2-diamide	99.0 ± 22.0	17.9 ± 5.3	0.78 ± 0.59	22.9	8.8 ± 0.48
VTD232	4-(2-methoxy-1-naphthyl)-6-phenyl-3,4-dihydro-2(1H)-pyrimidinethione	57.1 ± 30.9	30.5 ± 5.0	0.0039 ± 0.0046	7820	Not binding
VTD263	2-(4-ethoxyphenoxy)-N-[1-(5-ethyl-6-methyl-4-oxo(3-hydropyrimidin-2-yl))-3-methylpyrazol-5-yl]acetamide	100<	38.8 ± 0.76	11.4 ± 4.0	3.4	Not binding

^1^ CC_50_: 50% cytotoxic concentration, ^2^ IC_50_: 50% inhibition concentration, ^3^ SI: selective index, ^4^ Kd: dissociation constant.

## Data Availability

All data analyzed for the purposes of this study are included in this article.

## Supplementary Materials

The following supporting information can be downloaded at: https://www.mdpi.com/article/10.3390/v14061321/s1, Figure S1: Repeatability and dose dependency of Vpr-overexpressing yeast and the human ubiquitin-specific proteinase, Keap1 protein-overexpressing yeast, using 20, 2, and 0.2 μM of the compounds; Figure S2: Flag-Vpr expression in HeLa cells using Western blotting analysis; Figure S3: Localization of Vpr in HeLa cells, in the presence of 10 μM of three out of seven selected compounds, that suppresses Vpr-mediated G2/M arrest; Figure S4: Expression level of Vpr in HeLa cells, in the presence of 10 μM of three out of seven selected compounds, that suppresses Vpr-mediated G2/M arrest; Table S1: Results of the 1st screening for the new therapeutic drug, targeting HIV Vpr; Table S2: Results of the 2nd screening for the new therapeutic drug, targeting HIV Vpr; Table S3: Results of the 3rd screening for the new therapeutic drug, targeting HIV Vpr.
