# Supplementary material for: A Novel Class of HIV-1 Inhibitors Targeting the Vpr-Induced G2-Arrest in Macrophages by New Yeast- and Cell-Based High-Throughput Screening
Source: Viruses. 2022 Jun 16;14(6):1321. doi: 10.3390/v14061321 (PMC9227106; doi:10.3390/v14061321)
Supplement: Supplementary file 1 [file viruses-14-01321-s001.zip › Figures S1-S4.pdf]

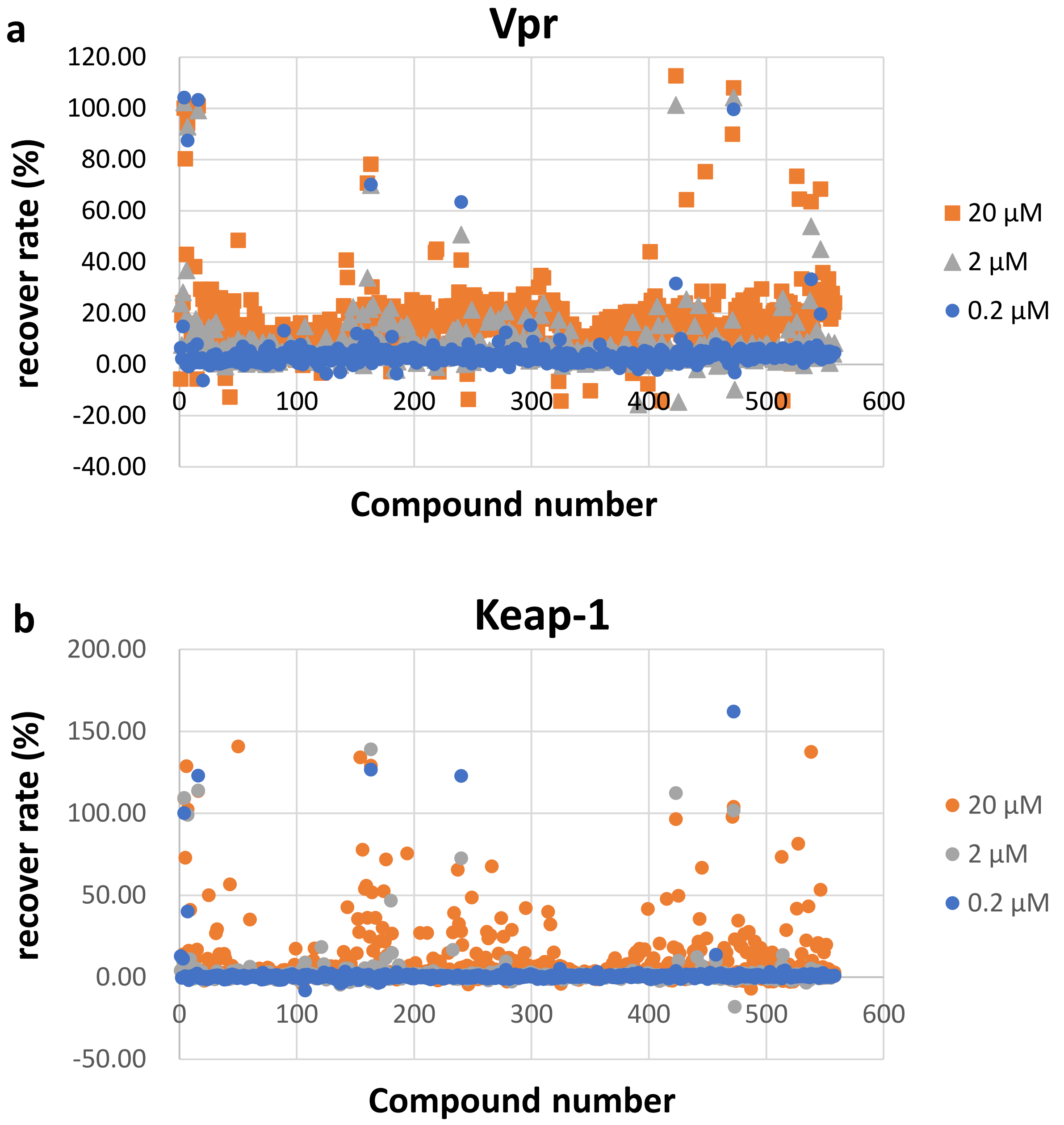

Figure S1: Repeatability and dose dependency of Vpr-overexpressing yeast (a) and the human ubiquitin-specific proteinase Keap1 protein-overexpressing yeast (b) using 20, 2, and 0.2 µM of compounds. All compounds were tested in twice, and the data presented are the mean recovery rate (%) of two experiments.

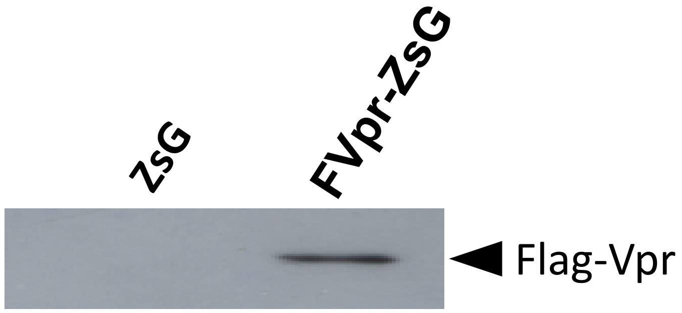

Figure S2: Flag-Vpr expression in HeLa cells using Western Blotting. HeLa cells were transfected with either pME/Flag-Vpr-IRES-ZsGreenI (FVpr-ZsG) or pME/Flag-IRES-ZsGreenI (ZsG), and then lysed. Lysates were subjected to western blotting analysis using anti-Flag M2 monoclonal antibody followed by HRP-conjugated goat anti-mouse IgG. The position of Flag-Vpr is indicated.

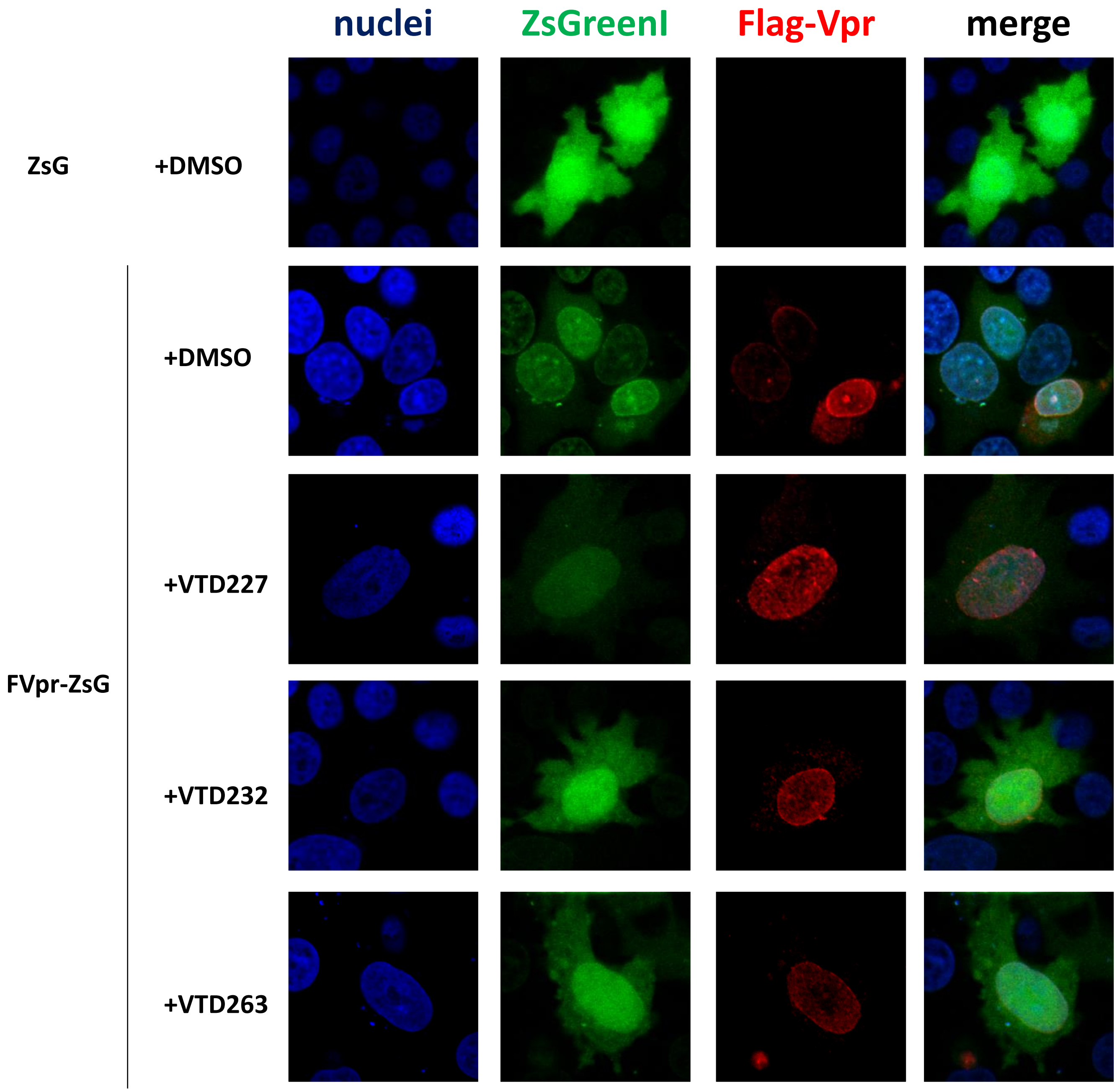

Figure S3: Localization of Vpr in HeLa cells in presence of 10 µM of three out of seven selected compounds that suppresses Vpr-mediated G2/M arrest. At 4 h post transfection with pME/Flag-Vpr-IRES-ZsGreenI (FVpr-ZsG) or pME/Flag-IRES-ZsGreenI (ZsG), HeLa cells were cultured in the absence or presence of three (VTD227, VTD232 and VTD263) out of seven representative compounds for 44 h; cells were harvested and subjected to immunofluorescence staining with Flag-specific M2 monoclonal antibody followed by Alexa Fluor 594-conjugated secondary antibody. The stained cells were visualized using an FV-1000 fluorescence microscope.

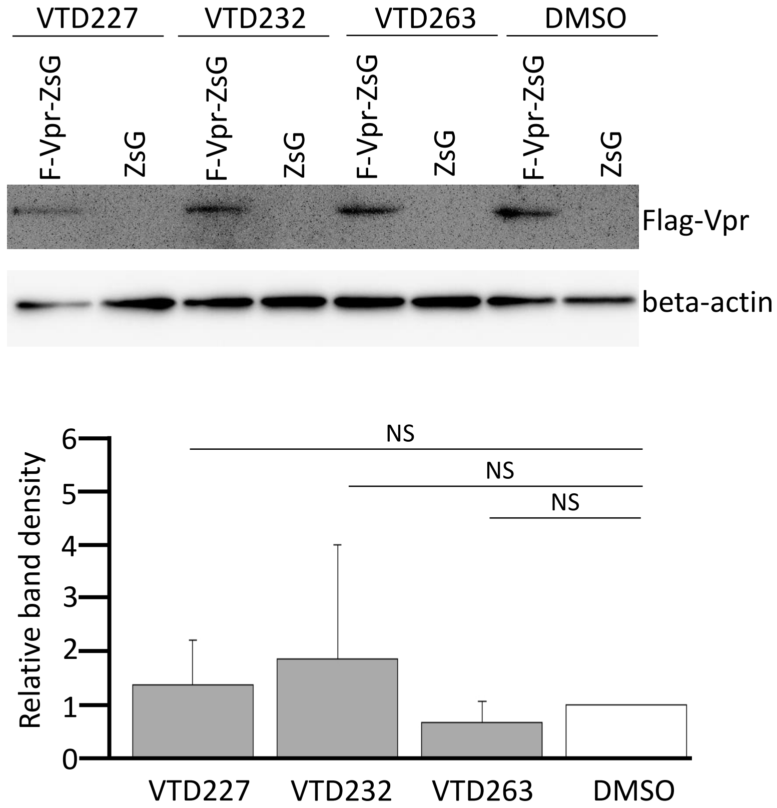

Figure S4: Expression level of Vpr in HeLa cells in presence of 10 μM of three out of seven selected compounds that suppresses Vpr-mediated G2/M arrest. At 4 h post transfection with pME/Flag-Vpr-IRES-ZsGreenI (FVpr-ZsG) or pME/Flag-IRES-ZsGreenI (ZsG), HeLa cells were cultured in the absence or presence of three (VTD227, VTD232 and VTD263) out of seven representative compounds for 44 h and cells were lysed. Lysates were applied to western blot using anti-FLAG M2 monoclonal antibody followed by HRP-conjugated goat anti-mouse IgG for detection of Flag-Vpr and anti-beta-actin monoclonal antibody followed by HRP-conjugated goat anti-mouse IgG for detection of for human beta-actin (upper panel). Band densities of Flag-Vpr and beta-actin were quantified by densitometry analysis using ImageJ software. The relative intensities were calculated as the ratio of density of Vpr to density of beta-actin (lower panel). Each column and error bar represents the mean ± SD for three independent experiments. Statistical comparisons were performed using Student's t-test. ns, not significant. The positions of Flag-Vpr and beta-actin are indicated.
